# Editorial: Non-invasive therapy for pain relief

**DOI:** 10.3389/fpain.2025.1690467

**Published:** 2025-09-10

**Authors:** Michael D. Staudt, Nader Pouratian, Jan Kubanek, Julie G. Pilitsis

**Affiliations:** ^1^ Department of Neurological Surgery, University Hospitals Cleveland Medical Center, Cleveland, OH, United States; ^2^ Case Western Reserve University School of Medicine, Cleveland, OH, United States; ^3^ Department of Neurological Surgery, UT Southwestern Medical Center, Dallas, TX, United States; ^4^ Department of Biomedical Engineering, University of Utah, Salt Lake City, UT, United States; ^5^ Department of Neurosurgery, University of Arizona College of Medicine, Tucson, AZ, United States

**Keywords:** chronic pain, focused ultrasound, neuromodulation, neuropathic pain, non-invasive, scrambler therapy, transcranial stimulation

**Editorial on the Research Topic**
Non-invasive therapy for pain relief

Chronic pain is prevalent and imposes significant suffering on patients, also placing a significant burden on the healthcare system (1). Furthermore, pain-related conditions are the primary reason that patients seek medical care or visit an emergency department in the United States (2). As such, the management of chronic pain can be exceedingly complex and requires a multimodal and multidisciplinary approach (3). Although there is no singular approach for the management of chronic pain syndromes, conservative therapies are initially preferred, with interventional procedures, neuromodulation, and surgery reserved for refractory pain; however, there is an unmet treatment need for a “middle ground” approach with non-invasive therapies. Non-invasive non-pharmacological therapies can be broadly categorized as physical modalities, psychological interventions such as cognitive-behavioral therapy, and complementary and alternative therapies, including acupuncture and yoga (4). Non-invasive neuromodulation therapies, such as transcranial magnetic stimulation (TMS), transcranial direct current stimulation (tDCS), and focused ultrasound (FUS), have emerged as promising alternatives to conventional treatments, although questions remain regarding their efficacy and durability. This special collection of articles aims to explore the advancements, clinical applications, and mechanistic insights of non-invasive therapies in pain management.

Non-invasive brain stimulation, including TMS and tDCS, has received much interest as a potential modality for modulating pain perception. Unlike TMS, which utilizes a magnetic field to stimulate specific brain regions, tDCS uses low-level electrical currents that are applied directly to the scalp. In a single-blinded, randomized, sham-controlled study, Tsai et al. evaluated pain detection thresholds in 53 healthy patients following active or sham tDCS. The anode and cathode were placed over the left primary sensorimotor cortex and the right dorsolateral prefrontal cortex, respectively, and were oriented either superior-medially or ventral-laterally. The authors found that the location and orientation of the anodal tDCS electrode influenced the modulation of pain sensitivity, as pain thresholds were increased when the connector was aligned superior-medially along the central sulcus.

A novel target for pain relief is the epidermis, which can serve as an interface for pain modulation via neuroimmune and endocrine signaling. Selva-Sarzo et al. evaluated the efficacy of transcutaneous neuromodulation applied to the lumbar spine to reduce pain in patients with chronic nonspecific low back pain. Their study followed a single-group crossover design, in which 39 patients underwent two interventions in a randomized sequence: transcutaneous neuromodulation tape with magnetic particles or a placebo with kinesiology tape. Transcutaneous neuromodulation applied to the lumbar spine significantly reduced perceived pain and increased ankle dorsiflexion range of motion compared to placebo, suggesting a potential role in the modulation of pain perception and motor function via interaction with epidermal afferents. This study demonstrated immediate improvements in pain relief and mobility; however, the long-term efficacy of this method is unclear and requires further investigation. Furthermore, comparisons with other non-invasive techniques are warranted, as are investigations into the potential influence of transcutaneous neuromodulation on pain processing at a cortical level.

FUS is a novel, non-invasive therapeutic technology with the potential to treat a wide range of neurological conditions. Currently approved clinical applications for intracranial use rely on high-intensity focused ultrasound for the purposes of ablation in the treatment of movement disorders. In contrast, low-intensity focused ultrasound (LIFU) is a non-destructive technique that can modulate neuronal activity without necessitating hardware implantation. Seol et al. presented a scoping review on the potential of LIFU for treating chronic pain and movement disorders. Chronic neuropathic pain is perhaps the most studied indication for LIFU, with the majority of preclinical studies focusing on targeting the lumbar dorsal root ganglia—many have demonstrated increased pain thresholds without histological evidence of tissue damage. Preclinical studies on the effects of LIFU on movement disorders and spinal cord injury are less common, although there is early evidence to support some suppression of tremors and spasticity. Due to the limited literature base, there is a knowledge gap regarding the optimal ultrasound parameters, and thus it is not possible to determine the relationship between treatment parameters and their effect on pain or spasticity/tremor modulation—this calls for standardization in reporting parameters and in the use of a common nomenclature.

The final article by Aboumerhi et al. reviewed the treatment of chemotherapy-induced peripheral neuropathy, which is a dose-limiting side effect of several cancer chemotherapeutic agents that can profoundly affect pain and function. The current standard of care relies primarily on neuropathic pain medications, with emerging evidence supporting the use of implantable neuromodulation devices such as spinal cord stimulators; however, there remains a significant unmet need for non-invasive or minimally invasive therapeutic modalities. Scrambler therapy is a non-invasive electro-analgesia treatment for chronic neuropathic and cancer-related pain. This technology requires the placement of cutaneous electrodes at the site of pain, followed by the algorithmic delivery of a low-intensity electrical current intended to mimic neuronal action potentials with continuously changing waveform patterns. The therapy is purported to work by disrupting the transmission of pain by replacing it with artificial pain-free signals. This review evaluated multiple observational and randomized studies and identified mixed results in terms of pain relief and the duration of its efficacy. As with many other non-invasive neuromodulation techniques for pain control, there is a lack of standardization in how the therapy is delivered, and studies are limited by low enrollment and short follow-up periods.

This research topic showcases some of the most recent advancements in chronic pain treatment using novel non-invasive neuromodulation therapies. As discussed, these therapies can be used to target different substrates, such as the brain, spine, and skin. Each area has different mechanisms to disrupt pain transmission at the central or peripheral level. There continues to be great interest in elucidating the mechanisms of pain transmission (5), and in targeting maladaptive circuitry (6). A critical question to ask when evaluating the efficacy of these therapies is, to what extent are they effective beyond the placebo effect? Outcomes in chronic pain treatment are notoriously prone to the placebo effect, with one meta-analysis reporting that non-speciﬁc effects accounted for approximately 78% of the improvement from invasive procedures in treating chronic pain conditions (7). This finding aligns with a common theme when evaluating non-invasive neuromodulation therapies—there is tremendous heterogeneity in treatment protocols and patient responses. Many studies have short-term follow-up, lack randomization or appropriate blinding, and are limited by small sample sizes. As such, the study of non-invasive neuromodulation therapies requires more rigor and standardization before appropriate guidelines for their use can be developed. Ultimately, their implementation in the treatment of chronic pain will likely be best served in a personalized, multi-modal approach.
